# Integrating Life Cycle Assessment in Innovative Berry Processing with Edible Coating and Osmotic Dehydration

**DOI:** 10.3390/foods14071167

**Published:** 2025-03-27

**Authors:** Alexandra Mari, Tryfon Kekes, Christos Boukouvalas, Magdalini Krokida

**Affiliations:** School of Chemical Engineering, National Technical University of Athens, Zografou, 15780 Athens, Greece

**Keywords:** Life Cycle Assessment, berries processing, sustainability, osmotic dehydration, edible coating

## Abstract

This study presents a Life Cycle Assessment (LCA) of a berry production system using osmotic dehydration and edible coating to extend the shelf life and improve the nutritional value. The goal is to evaluate environmental impacts, identify hotspots, and propose improvements. Osmotic dehydration is the main contributor to environmental impact, particularly due to the energy and resources required by apple juice as the osmotic agent. It contributes up to 0.64 kg CO_2_ eq. per kg of blueberries, 1.36 kg CO_2_ eq. per kg of raspberries, and 0.66 kg CO_2_ eq. per kg of strawberries. The edible coating, however, has minimal environmental impact due to its low energy consumption and biodegradable materials. Packaging has a lower carbon footprint but contributes more to fossil fuel depletion and human toxicity. Raspberries show the highest human health impact (3.5 × 10^−6^ DALY/kg) and ecosystem impact (9.5 × 10^−8^ species.yr/kg), followed by strawberries (1.78 × 10^−6^ DALY/kg, 4.97 × 10^−8^ species.yr/kg) and blueberries (1.7 × 10^−6^ DALY/kg, 5.1 × 10^−8^ species.yr/kg), highlighting the greater environmental and health costs of raspberries. Despite the environmental burden of osmotic dehydration, it offers economic benefits by extending the shelf life, reducing losses, improving supply chain efficiency, and enhancing product quality, which leads to higher prices and profit margins. The study concludes that, while the environmental impacts of osmotic dehydration should be optimized, its economic and logistical benefits make it a promising preservation solution. Further research into eco-friendly practices is recommended to reduce ecological costs while maintaining commercial advantages.

## 1. Introduction

Modern lifestyles are intricately linked to dietary habits, with fruits and vegetables being integral components of a healthy diet due to their abundance of bioactive compounds [1]. Berries are a valuable component of a healthy diet, known for their rich nutritional profile, including essential vitamins (C and K), minerals (manganese and potassium), and bioactive compounds such as flavonoids and anthocyanins [2]. These phytochemicals offer potent antioxidant and anti-inflammatory benefits, supporting heart health, brain function, and reducing the risk of chronic diseases like diabetes and cancer [3,4]. Due to their low-calorie content and natural sweetness, berries are increasingly popular among health-conscious consumers [5,6].

The shelf life of berries is a crucial factor in ensuring their availability and minimizing losses throughout the food supply chain. Due to their high perishability and rapid deterioration, berries are particularly susceptible to spoilage, which leads to significant food waste, especially during the transportation and storage stages [7]. The seasonal nature of berry production often results in periods of oversupply, which, if not efficiently managed, can lead to excess product that spoils before reaching consumers [8,9]. The losses incurred not only affect the economic viability of berry producers but also contribute to the broader environmental issue of food waste [10,11]. According to the United Nations Food and Agriculture Organization (FAO), 13.8% of global food production is lost annually before it even reaches consumers, with fruits and vegetables being particularly vulnerable, experiencing a loss rate of 21.6% [12]. This loss has profound economic implications, particularly in the berry industry, where oversupply and inadequate storage systems can drastically reduce profitability [13].

To mitigate these losses, various preservation techniques have been developed to extend the shelf life of berries while maintaining their quality and nutritional value. The ability to extend the freshness of berries provides significant economic benefits, reducing the volume of product waste and improving supply chain efficiency [5,14]. Preservation methods such as osmotic dehydration and edible coatings are gaining attention for their effectiveness in prolonging berry freshness and minimizing spoilage. Osmotic dehydration works by immersing berries in hypertonic solutions, which reduces the moisture content and inhibits microbial growth, thus slowing down spoilage and extending the shelf life [15]. Edible coatings, typically made from natural biopolymers such as polysaccharides, proteins, and lipids, create a protective barrier that limits moisture loss, prevents oxygen ingress, and reduces microbial contamination [16]. These coatings help maintain the quality and texture of berries, extending their freshness during storage and transportation [17,18]. Additionally, the extended shelf life made possible by these methods not only reduces food waste but also improves the overall efficiency of the berry supply chain, allowing for better inventory management, fewer transport-related losses, and greater profitability for producers [19].

Life Cycle Assessment (LCA) plays a crucial role in evaluating the environmental impacts of these preservation methods [20]. By examining the entire lifecycle of a product or process—from raw material extraction to disposal—LCA provides valuable insights into the resource use, energy consumption, and emissions associated with different preservation strategies [21]. In the case of osmotic dehydration and edible coatings, LCA can help determine their environmental sustainability by comparing their ecological footprint and identifying opportunities for improvement [22]. This comprehensive evaluation enables the identification of the most sustainable practices within the food industry, particularly in the context of berry preservation.

The aim of this study is to conduct an LCA of sustainable techniques for preserving the quality and extending the shelf life of berries. Specifically, the research analyzes the environmental impacts of osmotic dehydration and edible coatings as methods to prolong the shelf life of berries, with the primary goal being to assess the feasibility and sustainability of these technologies. This study takes a comprehensive approach to evaluating these preservation methods from an environmental perspective, focusing on their long-term sustainability and potential to reduce the ecological footprint of berry preservation.

## 2. Materials and Methods

Life Cycle Assessment (LCA) was performed following the guidelines outlined in ISO 14040 and 14044:2006 [23], and it consists of four steps: (i) Goal & Scope Definition, (ii) Inventory Analysis, (iii) Impact Assessment, and (iv) Interpretation.

### 2.1. Goal and Scope

The Goal of the LCA analysis was to determine the effect of the implementation of mild processing methods, such as osmotic dehydration and edible coatings, in the development of innovative berries with an increased shelf-life and high nutritional value.

A gate-to-gate approach was selected for the evaluation of the environmental footprint of processed berries. Specifically, the system boundaries encompass production processes from berries pickup to processing and packaging.

The data utilized in this study were sourced from the GaBi professional and Ecoinvent databases, which pertain to the geographical area of the European Union 28 (EU-28). All the studies and data collected are relevant to the past five years.

The Scope of the LCA analysis involves defining the goals and boundaries of the study, collecting data on resource inputs and environmental outputs throughout all stages of the life cycle, evaluating potential environmental impacts, and interpreting the results to guide decision-making and foster sustainability.

The Life Cycle Assessment (LCA) was performed following the recommendations proposed by the ISO 14040 recommendations series (14040:2006 and 14044:2006) [23]. ReCiPe 2016 (H, hierarchist) was selected as a method to perform the impact assessment, with its main objective being the transformation of Life Cycle Inventory results into a limited number of environmental impact scores using characterization factors. Finally, GaBi ts software (v10.6.2.9, Sphera Solutions GmbH, Echterdingen, Stuttgart, Germany) was used for the calculation of the impact categories [24].

The ReCiPe 2016 methodology defines impact indicators at two levels: midpoint and endpoint indicators. Midpoint indicators focus on specific environmental impacts, providing detailed insights into areas such as climate change, particulate matter formation, and resource depletion. These indicators cover issues like climate change (with and without biogenic carbon), particulate matter, mineral resource depletion, freshwater consumption and ecotoxicity, eutrophication, human toxicity (carcinogenic and non-carcinogenic), ionizing radiation, land use, marine ecotoxicity, photochemical ozone formation, stratospheric ozone depletion, and terrestrial acidification and ecotoxicity.

Endpoint indicators aggregate the midpoint indicators to simplify result interpretation. However, as aggregation increases, so does the uncertainty of the results. Endpoint indicators offer a broader overview of environmental impacts, assessing damage to human health (measured in Disability-Adjusted Life Years, DALY), ecosystems (measured in species.yr), and resource availability (measured in monetary terms, $).

#### 2.1.1. Product Systems and System’s Boundaries

**Figure 1:**
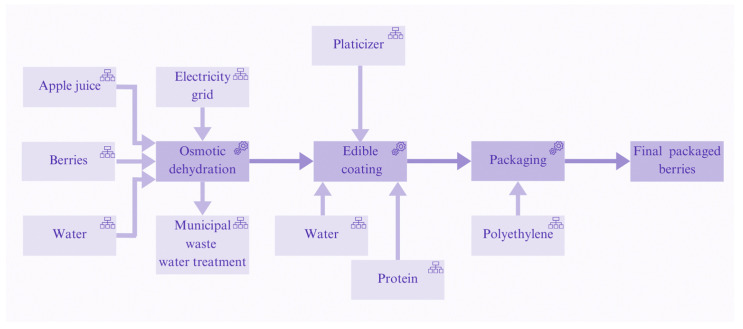
Flow chart of the innovative production of berries.

The evaluation was conducted on the innovative berry production line with extended shelf life. The system studied includes the process of receiving, processing, and packaging berries in an innovative production line, as shown in Figure 1. Specifically, compared to conventional berries that are received and directly packaged, processing methods of osmotic dehydration and edible coating have been added to the production phase.

#### 2.1.2. Process Analysis

**Figure 2:**
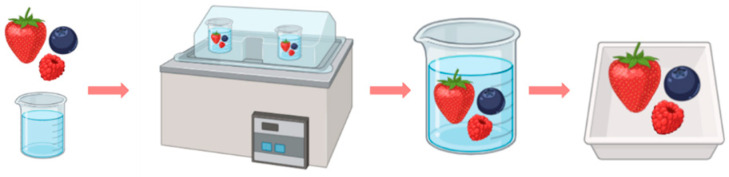
Process of the innovative production of berries.

Figure 2 provides a graphical representation of the production chain, illustrating the process from start to finish. It begins with the addition of freshly harvested berries into the osmotic solution, followed by their immersion in the edible coating solution, and concludes with the packaging of the berries as the final product.

#### Osmotic Dehydration

Osmotic dehydration is a natural and mild method of removing water from food using osmotic solutions. Based on the principle of osmosis, the process exploits the movement of water from a region of low solute concentration to a region of high solute concentration through a semipermeable membrane [18]. In the osmotic dehydration of berries, apple juice at 42°Brix has been selected as the optimum solution, after an optimization conducted in the laboratory regarding the dehydration kinetics, the quality characteristics of the final product, and the shelf life [25]. The duration of dehydration varies between the different types, with blueberries and raspberries at 360 min and strawberries at 200 min to achieve the optimal result. The process is carried out at 40 °C, maintaining the nutritional value and organoleptic characteristics of the berries and making it suitable for industrial application. This method helps preserve the natural texture, aroma, and flavor of the berries, as it does not require high temperatures or mechanical stresses that could damage the cell walls [26]. At the same time, osmotic dehydration reduces the need for preservatives and extensive processing, making the berries healthier and more appealing to consumers seeking naturally processed products. Finally, this method offers economic and environmental benefits, as it reduces energy and resource consumption, contributing to a sustainable and efficient production of products.

#### Edible Coating

Edible coating is an innovative technology applied in food processing, aimed at improving their quality and shelf life. This process involves coating the berries with an edible film made from natural materials such as polysaccharides, proteins, and lipids [27]. Edible coatings are designed to create a protective barrier that reduces moisture loss and prevents oxygen ingress while simultaneously protecting the berries from microbial contamination [28]. The edible coating was selected based on the evaluation of various coatings conducted by Mari et al. (2024) [29]. In their study, the coating derived from the protein of *Chlorella vulgaris* was identified as the most effective for enhancing the shelf life of berries. The system studied includes the process of receiving, processing, and packaging berries in an innovative production line, as shown in Figure 1. Specifically, compared to conventional berries that are received and directly packaged, processing methods of osmotic dehydration and edible coating have been added to the production phase.

#### Packaging

The berries were packaged using High-Density Polyethylene (HDPE) plastic, selected for its durability and protective qualities. The packaging was designed to minimize physical damage during handling, transportation, and storage. HDPE serves as an effective barrier against moisture, oxygen, and contaminants, helping to maintain the freshness, appearance, and quality of the berries over time. The material’s structural integrity ensures that the berries remain intact, reducing the risk of bruising or other damage [11]. The packaging was sealed to prevent moisture loss and exposure to air, thereby slowing down the deterioration process. HDPE’s transparent nature allows for easy visibility of the product, aiding in consumer decision-making while also providing a stable surface for labeling and branding. Furthermore, the choice of HDPE was based on its cost-effectiveness and widely recognized performance in extending the shelf life for perishable items [28,29,30,31].This packaging method is commonly used for fresh produce due to its effectiveness in protecting the product and facilitating handling and transport. However, the environmental impact of HDPE packaging was also considered, with a focus on exploring more sustainable alternatives for future applications.

#### 2.1.3. Functional Unit

The functional unit for the berry systems is defined as 1 kg (1 kg), while an analysis was also conducted using 1 euro (EUR) of revenue as a functional unit.

#### 2.1.4. Assumptions and Limitations

The data used for berry production in both cases is derived from experimental studies and supplemented with literature reviews to ensure accuracy and represent the current industry conditions. This study primarily aims to assess the environmental footprint of the proposed methods and evaluate their feasibility for managing solid and liquid waste. Additionally, energy consumption is carefully considered, particularly in the osmotic dehydration process, where the highest energy losses occur.

#### 2.1.5. Data Requirements

For the collection of data and the establishment of the inventory, values were obtained from experiments conducted by our research team between 2021 and 2024. These experimental data were combined with relevant literature data, and all figures were appropriately adjusted and verified through direct communication within the team.

### 2.2. Life Cycle Inventory

The Life Cycle Inventory (LCI) links processes with quantitative data based on the selected functional unit (1 kg of final packaged berries). Table 1, Table 2 and Table 3 present the input and output data for each process involved in berry processing, as depicted in Figure 2, for each berry. Literature and experimental data were used as a reference for data collection and inventory establishment, with appropriate adjustments made based on the specific context. These numbers were verified through careful review and consultation with relevant sources.

## 3. Results and Discussion

### 3.1. Life Cycle Impact Assessment

The results of the environmental impact assessment throughout the life cycle of the production of innovative berries (blueberry, raspberry, and strawberry) with extended shelf life, per 1 kg of final packaged berries, are presented in Table 4, Table 5 and Table 6.

**Figure 3:**
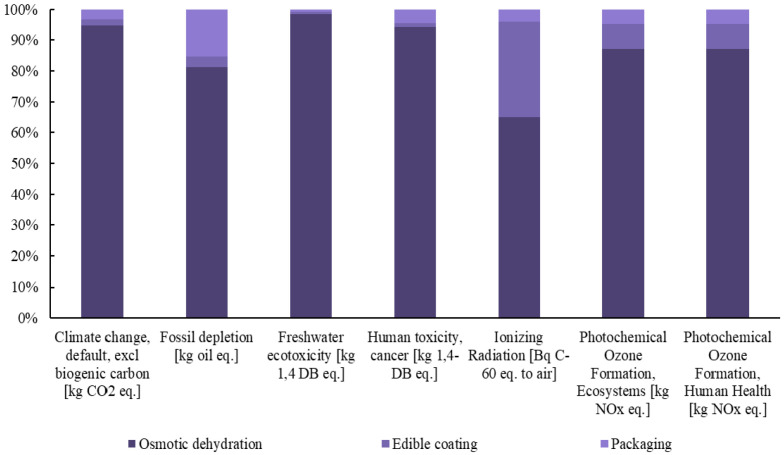
Contribution of each process to the final footprint of the selected midpoint impact categories for the production of innovative blueberries.

**Figure 4:**
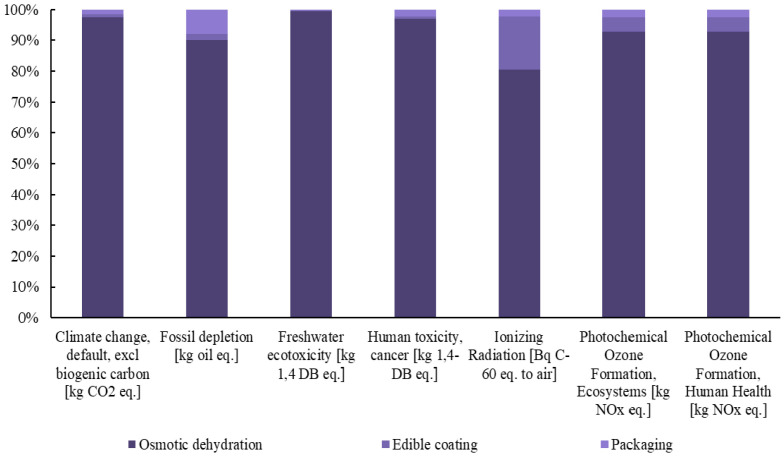
Contribution of each process to the final footprint of the selected midpoint impact categories for the production of innovative raspberries.

**Figure 5:**
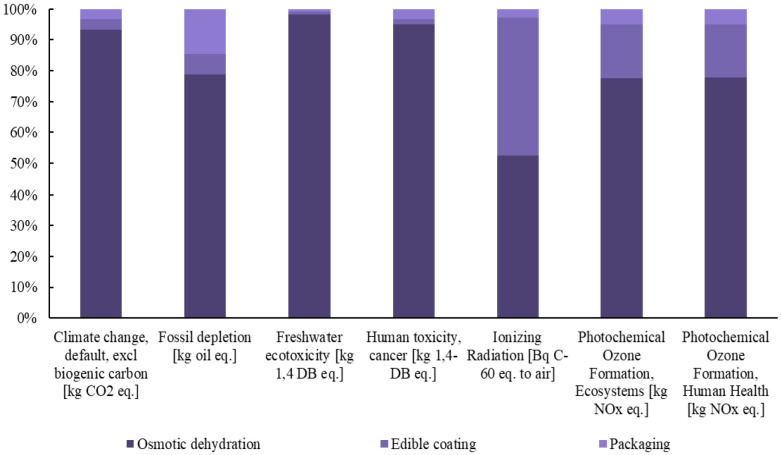
Contribution of each process to the final footprint of the selected midpoint impact categories for the production of innovative strawberries.

Subsequently, detailed diagrams are presented in Figure 3, Figure 4 and Figure 5, illustrating the contribution of each individual process to the total footprint of the selected midpoint impact categories, providing a comprehensive analysis of the environmental burden.

**Figure 6:**
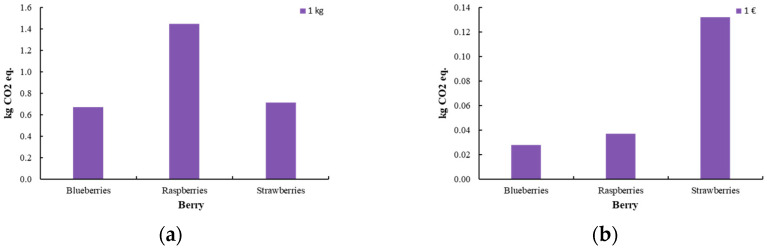
Climate change, default, and biogenic carbon [kg CO_2_ eq.] for the production of innovative berries (blueberry, raspberry, strawberry) (**a**) per 1 kg of product and (**b**) per 1 EUR of product.

Figure 6 represents the total climate change, default, and biogenic carbon of the berry process. The impact assessment was conducted using two functional units: product mass (1 kg) and economic value (1 EUR). This dual approach aims to evaluate the environmental and economic trade-offs, ensuring a comprehensive and well-founded analysis. The study investigates the environmental and socio-economic implications in relation to profit margins to derive robust and data-driven conclusions. The pricing of berries was determined based on an analysis of the Greek market, with the following estimated values: blueberries at 24 EUR/kg, raspberries at 39 EUR/kg, and strawberries at 5 EUR/kg.

**Figure 7:**
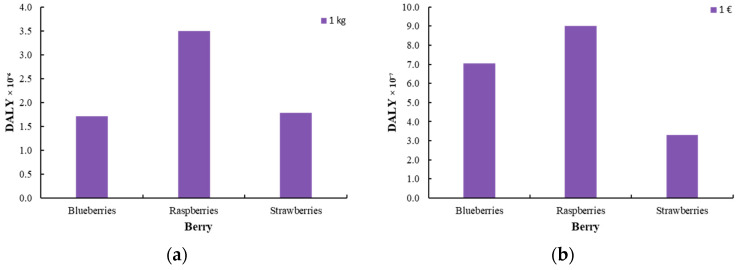
Damage to human health [DALY] for the production of innovative berries (blueberry, raspberry, and strawberry) (**a**) per 1 kg of product and (**b**) per 1 EUR of product.

**Figure 8:**
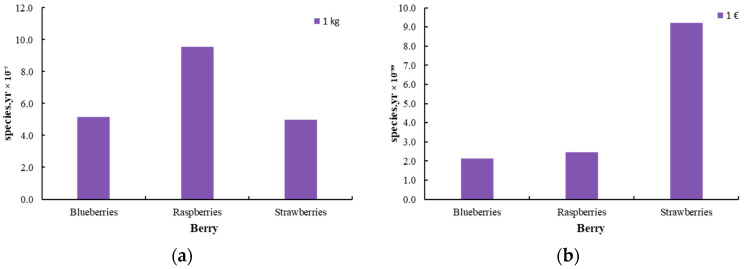
Damage to ecosystems [species.yr] for the production of innovative berries (blueberry, raspberry, and strawberry) (**a**) per 1 kg of product and (**b**) per 1 EUR of product.

**Figure 9:**
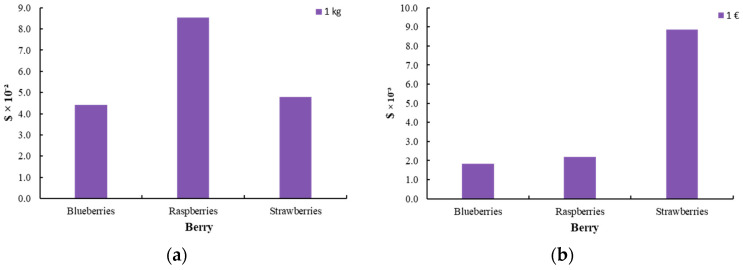
Damage to resource availability [$] for the production of innovative berries (blueberry, raspberry, and strawberry) (**a**) per 1 kg of product and (**b**) per 1 EUR of product.

Figure 7, Figure 8 and Figure 9 illustrate the endpoint impact categories, which describe the environmental consequences associated with the production of innovative berries. These impacts are classified into three main categories: effects on human health, ecosystem integrity, and resource availability. Human health impacts (DALY) quantify the potential risks arising from exposure to pollutants during berry processing. Ecosystem impacts (species.yr) assess potential biodiversity loss and ecosystem destabilization resulting from agricultural practices, while resource availability ($) reflects the depletion of natural resources and the corresponding economic costs associated with their use.

The impact assessment was carried out using two functional units to ensure a comprehensive evaluation: one based on product mass (1 kg) and the other on economic value (1 EUR). This dual approach allows for a more detailed analysis, considering both the physical quantity of the product and its financial significance in the market.

### 3.2. Interpretation

The Life Cycle Assessment (LCA) of the innovative berry production system, which integrates osmotic dehydration and edible coating techniques, offers critical insights into the environmental impact of these processing methods. This analysis highlights that osmotic dehydration is the primary driver of the increased environmental burden, contributing significantly to the environmental footprint across nearly all impact categories. Specifically, its contribution to climate change and land use is notably higher when compared to edible coating and packaging processes.

The use of osmotic dehydration significantly increases the environmental burden due to the use of apple juice as the osmotic agent, which has a substantial environmental footprint due to the cultivation and processing requirements of apples [32]. Furthermore, osmotic dehydration has been found to have detrimental environmental effects in other cases, particularly when alternative solvents such as glycerol are used. These solvents also contribute to heightened environmental impacts due to the energy required for their production, processing, and the waste generated during the dehydration process [33].

The environmental footprint of osmotic dehydration is not primarily attributed to electricity consumption. This indicates that, in terms of direct energy usage, the method is relatively energy-efficient compared to other dehydration techniques. However, the overall environmental impact stems mainly from the production and processing of osmotic agents rather than from the energy required to operate the dehydration process itself.

Despite these challenges, osmotic dehydration is widely used to extend the shelf life of fruit products, reducing spoilage and food waste [14,34,35]. A comprehensive assessment of its environmental benefits would require a full life cycle analysis, particularly evaluating its impact at the end-of-life stage. However, such studies have not yet been conducted due to a lack of sufficient data.

Opportunities exist for reducing the environmental footprint of osmotic dehydration. Recycling and reusing the osmotic solution through reverse osmosis technology is an important strategy for minimizing the environmental impact. Additionally, reducing the total volume of solvent required and optimizing the ratio of fruit to osmotic solution can further improve sustainability. Performing the process at temperatures close to ambient conditions can also yield significant energy savings, improving both energy efficiency and the overall sustainability of the method [36].

The edible coating process, on the other hand, does not contribute significantly to the environmental footprint, primarily because of the simplicity and sustainability of its production process. The raw materials used are biodegradable and plant-based, and the process requires minimal energy, particularly when performed at low temperatures. This helps reduce greenhouse gas emissions. Furthermore, the waste generated by edible coatings is minimal, as the residues are biodegradable and can be safely integrated into the environment. In addition to its low environmental impact, edible coating effectively extends the shelf life of food products by reducing moisture loss and slowing down microbial growth. Its environmental impact is almost insignificant in most categories, making it a highly sustainable food preservation technology and an environmentally friendly alternative to other food preservation methods [37,38].

Packaging, while still having a relatively low environmental impact, contributes more significantly to issues such as fossil fuel depletion and human toxicity, primarily due to the production and disposal of packaging materials [39]. In this process, the packaging remains the same as that used for fresh products, offering no additional environmental burden. However, sustainable and innovative packaging solutions, such as biodegradable or recyclable materials, could further enhance sustainability and should be explored in future research [40].

Despite the increased environmental burden, the innovative production method offers several advantages, most notably in extending the shelf life and improving the nutritional value of berries. These benefits, however, come with a higher environmental footprint compared to fresh berries, primarily due to the additional processing steps required. The overall environmental burden, particularly in terms of human health, resource availability, and ecosystem impacts, is higher for the processed berries, with osmotic dehydration and edible coating being the main contributors. Among the berries, raspberries exhibit the highest environmental footprint across all categories, which is attributed to the increased solvent use during osmotic dehydration, as optimized in the process. In contrast, the environmental impact of edible coating is consistent across all berry types, with no significant variations.

When the environmental footprint is normalized based on the selling price (EUR), significant differences in the impact of the preservation methods are observed. The environmental footprint of raspberries decreases and becomes more comparable to that of blueberries, while the footprint for strawberries increases substantially. This shift can be attributed to the higher commercial value of blueberries and raspberries, which justifies their relatively higher environmental impact despite the lower footprint observed in the analysis. The increased cost of strawberries, in comparison, amplifies their environmental footprint when normalized to price, reflecting the complexities of balancing economic value with environmental sustainability. On the other hand, the lower price of strawberries is insufficient to offset the environmental burden of the processing method, rendering it less efficient from both an environmental and economic perspective. However, if the extended shelf life of the product is taken into account, the overall environmental impact could be reconsidered, as reduced food waste may contribute to sustainability. Nevertheless, given that strawberry prices are not consistently low, the economic advantage remains limited, and the positive impact of the process is not significant.

While the environmental footprint of the innovative processing method is higher, the extended shelf life of the berries provides several economic and logistical benefits. The improved shelf life reduces losses during transportation and storage, enhancing supply chain efficiency. Furthermore, the ability to sell the berries at a higher price due to their extended shelf life increases profit margins and allows for distribution to high-demand markets and expanded geographical areas, thus improving commercial prospects.

Despite the increased environmental impact, the economic benefits derived from enhanced product preservation and reduced waste may mitigate some of the environmental costs. To further minimize the ecological footprint, it is essential to promote more environmentally friendly production practices while maintaining the commercial advantages offered by the extended shelf life of innovative berries.

## 4. Conclusions

In conclusion, the Life Cycle Assessment (LCA) of the innovative berry production system reveals that, while osmotic dehydration significantly contributes to the environmental footprint, the overall environmental impact can be mitigated through various strategies. The primary environmental burden stems from the use of apple juice as the osmotic agent, with its cultivation and processing contributing to high environmental costs. However, opportunities for reducing these impacts exist, such as optimizing the use of solvents, implementing reverse osmosis for solution recycling, and conducting the process at lower temperatures to save energy. On the other hand, edible coating and packaging demonstrate relatively low environmental footprints, with edible coating in particular proving to be a highly sustainable preservation method due to its low energy demand and minimal waste generation. To reduce the overall environmental burden, it is essential to refine the osmotic dehydration process and further explore sustainable packaging solutions.

Despite the higher environmental footprint compared to fresh berries, the innovative processing methods offer substantial economic and logistical benefits. The extended shelf life achieved through osmotic dehydration and edible coating reduces losses during transportation and storage, enhancing the supply chain efficiency and allowing berries to be sold at higher prices. This not only increases profit margins but also enables distribution to more markets, expanding commercial opportunities. Therefore, while the environmental challenges associated with these processing techniques should not be overlooked, the economic advantages, along with strategies to minimize ecological impacts, offer a pathway toward more sustainable and profitable berry production. Emphasizing environmentally friendly production practices and optimizing the entire production process will be key to ensuring the sustainability of this innovative berry preservation approach.

## Figures and Tables

**Table 1:** Life Cycle Inventory (LCI) for innovative blueberries, expressed on a unit basis (1 kg of final packaged berries) of the final blueberry product.

Process	Flow	Quantity
Osmotic dehydration	[In] Blueberries (kg)	0.91
	[In] Apple juice (kg)	1.20
	[In] Water (kg)	1.02
	[In] Electricity (MJ)	0.25
	[Out] Blueberries (kg)	0.90
	[Out] Wastewater (kg)	1.72
Edible coating	[In] Berries (kg)	0.90
	[In] Protein (kg)	0.01
	[In] Tween 20 (kg)	3.58 × 10^−4^
	[In] Glycerol (kg)	2.69 × 10^−3^
	[In] Water (kg)	0.08
	[Out] Blueberries (kg)	0.99
Packaging	[In] Blueberries (kg)	0.99
	[In] HDPE (kg)	0.01
	[Out] Blueberries (kg)	1.00

**Table 2:** Life Cycle Inventory (LCI) for innovative raspberries, expressed on a unit basis (1 kg of final packaged berries) of the final raspberry product.

Process	Flow	Quantity
Osmotic dehydration	[In] Raspberries (kg)	1.17
	[In] Apple juice (kg)	1.52
	[In] Water (kg)	1.30
	[In] Electricity (MJ)	0.20
	[Out] Raspberries (kg)	0.90
	[Out] Wastewater (kg)	2.60
Edible coating	[In] Berries (kg)	0.90
	[In] Protein (kg)	0.01
	[In] Tween 20 (kg)	3.58 × 10^−4^
	[In] Glycerol (kg)	2.69 × 10^−3^
	[In] Water (kg)	0.08
	[Out] Raspberries (kg)	0.99
Packaging	[In] Raspberries (kg)	0.99
	[In] HDPE (kg)	0.01
	[Out] Raspberries (kg)	1.00

**Table 3:** Life Cycle Inventory (LCI) for innovative strawberries, expressed on a unit basis (1 kg of final packaged berries) of the final strawberry product.

Process	Flow	Quantity
Osmotic dehydration	[In] Strawberries (kg)	1.03
	[In] Apple juice (kg)	0.75
	[In] Water (kg)	1.15
	[In] Electricity (MJ)	0.25
	[Out] Strawberries (kg)	0.90
	[Out] Wastewater (kg)	3.94
Edible coating	[In] Berries (kg)	0.90
	[In] Protein (kg)	0.01
	[In] Tween 20 (kg)	3.58 × 10^−4^
	[In] Glycerol (kg)	2.69 × 10^−3^
	[In] Water (kg)	0.08
	[Out] Strawberries (kg)	0.99
Packaging	[In] Strawberries (kg)	0.99
	[In] HDPE (kg)	0.01
	[Out] Strawberries (kg)	1.00

**Table 4:** Life cycle impact assessment results for the innovative blueberries (per 1 kg of final packaged berries) for the selected conventional and innovative midpoint impact categories.

Midpoint Impact Categories	Units	Osmotic Dehydration	Edible Coating	Packaging	Total
Climate change, default, excl biogenic carbon	kg CO_2_ eq.	6.35E−01	1.27E−02	2.26E−02	6.70E−01
Climate change, incl biogenic carbon	kg CO_2_ eq.	4.02E−01	4.21E−03	2.27E−02	4.29E−01
Fine Particulate Matter Formation	kg PM_2.5_ eq.	6.42E−04	1.01E−05	7.88E−06	6.60E−04
Fossil depletion	kg oil eq.	1.20E−01	5.01E−03	2.28E−02	1.48E−01
Freshwater Consumption	m^3^	6.92E−02	1.13E−04	9.86E−05	6.94E−02
Freshwater ecotoxicity	kg 1.4 DB eq.	1.03E−03	6.94E−06	8.24E−06	1.05E−03
Freshwater Eutrophication	kg P eq.	1.09E−05	1.08E−06	3.46E−08	1.20E−05
Human toxicity, cancer	kg 1.4-DB eq.	2.80E−04	3.79E−06	1.35E−05	2.97E−04
Human toxicity, non-cancer	kg 1.4-DB eq.	2.66E−02	1.27E−02	2.83E−03	4.21E−02
Ionizing Radiation	Bq C-60 eq. to air	1.29E−03	6.14E−04	7.77E−05	1.98E−03
Land use	Annual crop eq.·y	3.56E−01	1.31E−02	2.94E−04	3.69E−01
Marine ecotoxicity	kg 1.4-DB eq.	8.64E−04	1.11E−05	2.44E−05	9.00E−04
Marine Eutrophication	kg N eq.	4.29E−04	8.28E−06	2.86E−07	4.38E−04
Metal depletion	kg Cu eq.	5.35E−04	3.17E−04	1.63E−05	8.68E−04
Photochemical Ozone Formation, Ecosystems	kg NO_x_ eq.	2.99E−01	2.82E−02	1.62E−02	3.43E−01
Photochemical Ozone Formation, Human Health	kg NO_x_ eq.	1.87E−01	1.75E−02	1.00E−02	2.15E−01
Stratospheric Ozone Depletion	kg CFC-11 eq.	1.67E−06	2.18E−08	4.88E−09	1.70E−06
Terrestrial Acidification	kg SO_2_ eq.	1.88E−03	4.35E−05	2.37E−05	1.95E−03
Terrestrial ecotoxicity	kg 1.4-DB eq.	7.14E−02	5.25E−03	3.12E−03	7.98E−02

**Table 5:** Life cycle impact assessment results for the innovative raspberries (per 1 kg of final packaged berries) for the selected conventional and innovative midpoint impact categories.

Midpoint Impact Categories	Units	Osmotic Dehydration	Edible Coating	Packaging	Total
Climate change, default, excl biogenic carbon	kg CO_2_ eq.	1.36E+00	6.36E−02	2.26E−02	1.45E+00
Climate change, incl biogenic carbon	kg CO_2_ eq.	8.35E−01	2.14E−02	2.26E−02	8.79E−01
Fine Particulate Matter Formation	kg PM_2.5_ eq.	1.39E−03	5.05E−05	7.86E−06	1.45E−03
Fossil depletion	kg oil eq.	2.56E−01	2.51E−02	2.27E−02	3.04E−01
Freshwater Consumption	m^3^	1.55E−01	5.67E−04	9.83E−05	1.56E−01
Freshwater ecotoxicity	kg 1.4 DB eq.	2.27E−03	3.45E−05	8.22E−06	2.31E−03
Freshwater Eutrophication	kg P eq.	2.24E−05	5.39E−06	3.45E−08	2.78E−05
Human toxicity, cancer	kg 1.4-DB eq.	5.53E−04	1.90E−05	1.35E−05	5.86E−04
Human toxicity, non-cancer	kg 1.4-DB eq.	5.67E−02	6.27E−02	2.82E−03	1.22E−01
Ionizing Radiation	Bq C-60 eq. to air	2.86E−03	3.07E−03	7.74E−05	6.01E−03
Land use	Annual crop eq.·y	7.92E−01	6.52E−02	2.93E−04	8.57E−01
Marine ecotoxicity	kg 1.4-DB eq.	1.89E−03	5.53E−05	2.44E−05	1.97E−03
Marine Eutrophication	kg N eq.	9.53E−04	4.12E−05	2.85E−07	9.94E−04
Metal depletion	kg Cu eq.	1.12E−03	1.58E−03	1.63E−05	2.72E−03
Photochemical Ozone Formation, Ecosystems	kg NO_x_ eq.	5.78E−01	1.41E−01	1.61E−02	7.35E−01
Photochemical Ozone Formation, Human Health	kg NO_x_ eq.	3.61E−01	8.77E−02	1.00E−02	4.59E−01
Stratospheric Ozone Depletion	kg CFC-11 eq.	3.71E−06	1.08E−07	4.86E−09	3.82E−06
Terrestrial Acidification	kg SO_2_ eq.	4.09E−03	2.16E−04	2.36E−05	4.33E−03
Terrestrial ecotoxicity	kg 1.4-DB eq.	1.29E−01	2.61E−02	3.11E−03	1.58E−01

**Table 6:** Life cycle impact assessment results for the innovative strawberries (per 1 kg of final packaged berries) for the selected conventional and innovative midpoint impact categories.

Midpoint Impact Categories	Units	Osmotic Dehydration	Edible Coating	Packaging	Total
Climate change, default, excl biogenic carbon	kg CO_2_ eq.	6.65E−01	2.55E−02	2.27E−02	7.13E−01
Climate change, incl biogenic carbon	kg CO_2_ eq.	4.15E−01	8.51E−03	2.27E−02	4.46E−01
Fine Particulate Matter Formation	kg PM_2.5_ eq.	6.75E−04	2.02E−05	7.90E−06	7.03E−04
Fossil depletion	kg oil eq.	1.23E−01	1.01E−02	2.29E−02	1.56E−01
Freshwater Consumption	m3	7.41E−02	2.27E−04	9.88E−05	7.44E−02
Freshwater ecotoxicity	kg 1.4 DB eq.	1.15E−03	1.39E−05	8.26E−06	1.17E−03
Freshwater Eutrophication	kg P eq.	1.51E−05	2.16E−06	3.47E−08	1.73E−05
Human toxicity, cancer	kg 1.4-DB eq.	4.03E−04	7.60E−06	1.36E−05	4.24E−04
Human toxicity, non-cancer	kg 1.4-DB eq.	2.81E−02	2.53E−02	2.84E−03	5.62E−02
Ionizing Radiation	Bq C-60 eq. to air	1.44E−03	1.23E−03	7.78E−05	2.75E−03
Land use	Annual crop eq.·y	3.92E−01	2.61E−02	2.94E−04	4.18E−01
Marine ecotoxicity	kg 1.4-DB eq.	9.60E−04	2.22E−05	2.45E−05	1.01E−03
Marine Eutrophication	kg N eq.	4.78E−04	1.65E−05	2.87E−07	4.95E−04
Metal depletion	kg Cu eq.	6.25E−04	6.33E−04	1.64E−05	1.27E−03
Photochemical Ozone Formation, Ecosystems	kg NO_x_ eq.	2.52E−01	5.65E−02	1.62E−02	3.25E−01
Photochemical Ozone Formation, Human Health	kg NO_x_ eq.	1.58E−01	3.51E−02	1.01E−02	2.03E−01
Stratospheric Ozone Depletion	kg CFC-11 eq.	1.83E−06	4.35E−08	4.89E−09	1.88E−06
Terrestrial Acidification	kg SO_2_ eq.	1.98E−03	8.69E−05	2.37E−05	2.09E−03
Terrestrial ecotoxicity	kg 1.4-DB eq.	4.81E−02	1.05E−02	3.13E−03	6.17E−02

## Data Availability

The original contributions presented in this study are included in the article. Further inquiries can be directed to the corresponding author.

## Abbreviations

LCA—Life Cycle Assessment, ISO—International Organization for Standardization, EU-28—European Union (28 member states), HDPE—High-Density Polyethylene, MJ—Megajoule, CO_2_ eq.—Carbon Dioxide Equivalent, PM2.5 eq.—Particulate Matter (diameter ≤ 2.5 µm) Equivalent, DB eq.—1,4-Dichlorobenzene Equivalent (used in toxicity assessments), P eq.—Phosphorus Equivalent, N eq.—Nitrogen Equivalent, Cu eq.—Copper Equivalent, NOx eq.—Nitrogen Oxides Equivalent, CFC-11 eq.—Trichlorofluoromethane Equivalent (Ozone Depleting Substance), SO_2_ eq.—Sulfur Dioxide Equivalent, Bq C-60 eq. to air—Becquerel of Cobalt-60 Equivalent to Air (Ionizing Radiation Indicator), DALY—Disability-Adjusted Life Years (used for Human Health Impact), GaBi—Software used for Life Cycle Analysis (by Sphera Solutions), ReCiPe—A life cycle impact assessment method, FAO—Food and Agriculture Organization, CE—Circular Economy
